# The effects of different doses of dexmedetomidine on the requirements for propofol for loss of consciousness in patients monitored via the bispectral index: a double-blind, placebo-controlled trial

**DOI:** 10.1186/s12871-020-01013-x

**Published:** 2020-04-25

**Authors:** Yang Gu, Fan Yang, Yonghai Zhang, Junwei Zheng, Jie Wang, Bin Li, Tao Ma, Xiang Cui, Kaimei Lu, Hanxiang Ma

**Affiliations:** 1 Department of Anesthesiology, Ningxia Medical University, Yinchuan, 750004 China; 2 Department of Anesthesiology, People’s Hospital of Ningxia Hui Autonomous Region, Yinchuan, 750002 China; 3 Department of Anesthesiology, General Hospital of Ningxia Medical University, Yinchuan, 750004 China

**Keywords:** Propofol, Dexmedetomidine, Bispectral index, Loss of consciousness

## Abstract

**Background:**

The α_2_-adrenergic agonist dexmedetomidine (DEX) is a sedative and can be used as an adjunct to hypnotics. The study sought to evaluate the effects of different doses of DEX on the requirements for propofol for loss of consciousness (LOC) in patients monitored via the bispectral index (BIS).

**Methods:**

In this randomized, double-blind, three arm parallel group design and placebo-controlled trial, 73 patients aged between 18 and ~ 65 years with a BMI range of 18.0–24.5 kg·m^− 2^ and an American Society of Anesthesiologists (ASA) grade I or II who were scheduled for general anesthesia at the General Hospital of Ningxia Medical University were included in this study. Anesthesiologists and patients were blinded to the syringe contents. All patients were randomly assigned in a 1:1:1 ratio to receive a 0.5 μg·kg^− 1^ DEX infusion (0.5 μg·kg^− 1^ DEX group; *n* = 24), a 1.0 μg·kg^− 1^ DEX infusion (1.0 μg·kg^− 1^ DEX group; *n* = 25) or a saline infusion (control group; *n* = 24) for 10 min. Propofol at a concentration of 20 mg·kg^− 1^·h^− 1^ was then infused at the end of the DEX or saline infusion. The propofol infusion was stopped when the patient being infused lost consciousness. The primary endpoint were propofol requirements for LOC and BIS value at LOC.

**Results:**

The data from 73 patients were analyzed. The propofol requirements for LOC was reduced in the DEX groups compared with the control group (1.12 ± 0.33 mg·kg^− 1^ for the 0.5 μg·kg^− 1^ DEX group vs. 1.79 ± 0.39 mg·kg^− 1^ for the control group; difference, 0.68 mg·kg^− 1^ [95% CI, 0.49 to 0.87]; *P* = 0.0001) (0.77 ± 0.27 mg·kg^− 1^ for the 1.0 μg·kg^− 1^ DEX group vs. 1.79 ± 0.39 mg·kg^− 1^ for the control group; difference, 1.02 mg·kg^− 1^ [95% CI, 0.84 to 1.21]; *P* = 0.0001). The propofol requirements for LOC was lower in the 1.0 μg·kg^− 1^ DEX group than the 0.5 μg·kg^− 1^ DEX group (0.77 ± 0.27 mg·kg^− 1^ vs. 1.12 ± 0.33 mg·kg^− 1^, respectively; difference, 0.34 mg·kg^− 1^ [95% CI, 0.16 to 0.54]; *P* = 0.003). At the time of LOC, the BIS value was higher in the DEX groups than in the control group (67.5 ± 3.5 for group 0.5 μg·kg^− 1^ DEX vs. 60.5 ± 3.8 for the control group; difference, 7.04 [95% CI, 4.85 to 9.23]; *P* = 0.0001) (68.4 ± 4.1 for group 1.0 μg·kg^− 1^ DEX vs. 60.5 ± 3.8 for the control group; difference, 7.58 [95% CI, 5.41 to 9.75]; *P* = 0.0001).

**Conclusion:**

The study showed that DEX (both 0.5 and 1.0 μg·kg^− 1^ DEX) reduced the propofol requirements for LOC. DEX pre-administration increased the BIS value for LOC induced by propofol.

**Clinical trial registration:**

The study was registered at ClinicalTrials.gov (trial ID: NCT02783846 on May 26, 2016).

## Background

A variety of sedatives, such as propofol combined with midazolam, are commonly used in induction of anesthesia. Dexmedetomidine (DEX) is now commonly used in anesthesia induction because of its sympatholytic effect and it can attenuate the cardiovascular response during intubation [1, 2]. However, there is still a lack of clinical experience in the combined use of these two drugs in the induction of anesthesia, and sedative overdose may occur during induction of anesthesia.

Along with other drugs, propofol is frequently used as a sedative-hypnotic drug to induce anesthesia. Unfortunately, propofol at the recommended induction dose (2.0–2.5 mg·kg^− 1^) often causes cardiovascular depression during anesthesia induction [3]. Theoretically, decreasing propofol dose is associated with a low incidence of hypotension during anesthesia induction [4]. The techniques decreasing propofol dose for anesthesia induction as guided by bispectral index [5] may reduce the incidence of hypotension induced by recommended propofol dose.

DEX is a widely used drug in anesthesia for its sympatholytic, sedative and analgesic effects [6]. It has been reported that DEX decreased the propofol requirements by bispectral index-guided closed-loop anesthesia [7]. Many studies have observed the opioid-free effect of DEX when combined with other drugs during anesthesia [8, 9]. But few study has focused on the effect of DEX on propofol requirements for loss of consciousness (LOC) during anesthesia induction. Indeed, anesthesia induction is an important phase in the perioperative period; thus, it is urgent to separately evaluate the effect of DEX on the propofol requirements for LOC during this phase. Considering the anesthetic-free effect of DEX, we hypothesized that DEX can decrease the propofol requirements for LOC during anesthesia induction. Therefore, the first purpose of this study was to verify that DEX decreases the propofol requirements for LOC during anesthesia induction.

The bispectral index (BIS) is a common tool to determine the depth of the sedative state. The BIS value is constantly maintained ranged from 40 to 60 during general anesthesia through the titration of anesthetic agents [10]. It had been demonstrated that there was a good relationship between the BIS values and the blood concentration of propofol at LOC [11]. However, the BIS value at LOC varies when different sedatives are used [12]. It has been proven that the BIS value was different at the loss of response to voice commands when fentanyl, nitrous oxide, or alfentanil was added to the propofol anesthesia [13]. DEX produced resembling stage 2 NREM sleep in the EEG and characteristic arousal sedation [14, 15], and these makes it distinguishes from propofol. Therefore, we hypothesized that the BIS value at LOC was different between propofol alone and propofol combined with DEX administration. Thus, the second goal of this study was to evaluate the effect of DEX on the BIS value at LOC induced by propofol.

## Methods

The study was approved by the ethics committee of the General Hospital of Ningxia Medical University (2016167). The study was registered at ClinicalTrials.gov (NCT02783846). The study was conducted in accordance with applicable CONSORT guidelines. This was a prospective, double blind, single center randomized study with a three arm parallel group design. No changes were made regarding important changes to methods after trial commencement. Written informed consent was obtained from 87 patients with an American Society of Anesthesiologists (ASA) score I or II, an age of 18–65 years, and a body mass index (BMI) of 18.0–24.5 kg·m^− 2^ who were scheduled for elective surgeries under general anesthesia. Exclusion criteria included an allergy to α_2_-adrenergic agonists, bradycardia, atrioventricular block, neurologic disorders and the recent use of psychoactive medications, hearing impairment, or alcohol abuse.

Sample size estimation was performed using NCSS-PASS software (version 11.0.7, Update time 2013-01-22). In a one-way ANOVA study, we estimated that the sample sizes of 0.5 μg·kg^− 1^ DEX group, 1 μg·kg^− 1^ DEX group and control group were 22, 23, and 22, which means were to be compared. The total sample of 67 subjects achieves 90% power to detect differences among the means versus the alternative of equal means using an F test with a 0.05 significance level. The data of the pilot study were not included in data analysis in the current study. Given an anticipated dropout rate of 10%, a total of 73 patients (*n* = 73) were incorporated in the study and distributed randomly with a 1:1:1 ratio into three groups: the 0.5 μg·kg^− 1^ DEX group (*n* = 24), the 1.0 μg·kg^− 1^ DEX group (*n* = 25) and the control group (*n* = 24), respectively. No interim analysis were made.

A computer-generated randomization table was used to assign each patient to one of the three groups. Study drugs (DEX or normal saline) in the identical 50-ml syringes were prepared by a pharmacist, and the syringes were consecutively numbered according to the randomization schedule. The details were as follows: the solution administered to the 1.0 μg·kg^− 1^ DEX group was prepared by dissolving one ampoule of DEX (containing 200 μg in a concentration of 100 μg·ml^− 1^) in normal saline to make a 50 ml solution, yielding a final concentration of 4 μg·ml^− 1^; the solution administered to the 0.5 μg·kg^− 1^ DEX group was prepared by dissolving one-half of an ampoule of DEX in normal saline to make a 50 ml solution, yielding a final concentration of 2 μg·ml^− 1^; for the solution administered to the patients of the control group, only 50 ml of 0.9% saline was prepared. Each patient was assigned an order number and received the different drugs, and the anesthesiologists were blinded to the syringe contents. No changes were made regarding blinding. DEX (100 μg·ml^− 1^) and propofol (10 mg·ml^− 1^) were supplied by Sichuan Guorui Medicine Co. Ltd. (Sichuan, China) in identical 2-ml ampules and AstraZeneca Corporation (London, England) in identical 50-ml ampules, respectively.

Patients were admitted to the operating room with no pre-medication. An 18G catheter was inserted into the large forearm vein for fluid and drug administration. Lactated Ringer’s solution was infused at a rate of 15 ml·kg^− 1^·h^− 1^ before the study. Non-invasive arterial pressure, electrocardiogram, and peripheral oxygen saturation (SpO_2_) were continuously measured throughout the study period. The BIS was derived from the frontal electroencephalogram and calculated by an Aspect Vista monitor (version 3.2, Aspect Medical System, Inc.) using BIS sensor electrodes. Four cutaneous electrodes (ZipPrep; Aspect) were positioned: At_1_ and At_2_ (one each above the outer malar bones) with Fp (4 cm above the nasion) as the reference and Fp_2_ (left forehead) as the ground. Impedance was kept at < 2000 Ω. The BIS (100 = awake, 0 = burse suppression) and its trend were displayed continuously. The time delay of the BIS should be addressed; therefore, the BIS data were recorded after the propofol infusion reached 61 s [16].

All groups were infused with a loading dose of DEX or normal saline via a Graseby syringe pump model 3500 at a speed of 1.5 ml·kg^− 1^·h^− 1^ for 10 min. After the loading doses of DEX or normal saline, the propofol was not stopped with a continuous intravenous infusion by micro-pump at 20 mg·kg^− 1^·h^− 1^ until the patient lost consciousness. The state of consciousness was evaluated once the propofol was initiated, with an interval of 10 s, until the patients lost consciousness. The endpoint of LOC was determined by loss of the eyelash reflex and not responded to their own name called loudly and repeatedly.

The primary outcomes were the propofol requirements for LOC and the BIS value at LOC. The secondary outcomes was the time to LOC. The mean arterial pressure (MAP), heart rate (HR) and the BIS value were recorded before infusion of the study drug, with an interval of 5 min throughout the study period. No changes were made regarding trial outcomes after the trial commenced.

If the systolic arterial blood pressure increased or decreased by 20% from the baseline or the systolic pressure was less than 90 mmHg, the urapidil or phenylephrine was administered immediately to adjust the blood pressure within a normal range. The atropine was used to maintain the HR above 50 beats·min^− 1^. Respiratory depression was treated with assisted ventilation via facemask.

The data were recorded using Microsoft Excel 2007 and analyzed with various statistical tests using SPSS 17.0 (SPSS Inc., Chicago, IL, USA). Sex distribution was analyzed using the chi-square (Х^2^) test. The propofol requirements for LOC, the BIS values at LOC, the time to LOC, and the patients’ characteristics (age, height, and weight) were analyzed using analysis of variance (ANOVA) and LSD multiple comparisons. Statistical significance was defined as a *P* value of less than 0.05.

## Results

**Fig. 1:**
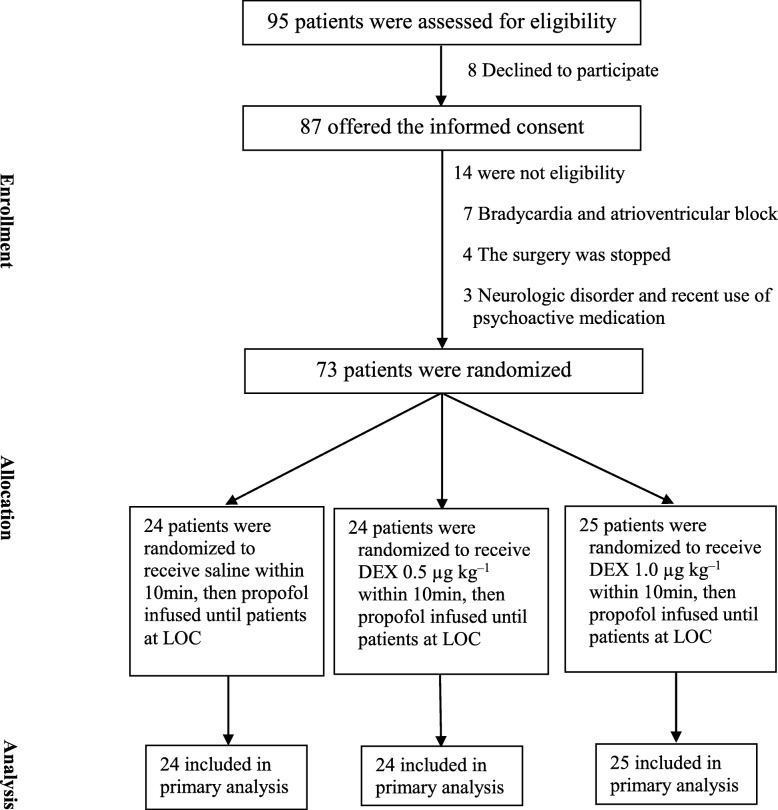
Patient-flow diagram

The trial was conducted from June 16, 2016 to August 17, 2016 at the General Hospital of Ningxia Medical University. A total of 73 patients were ultimately enrolled. Twenty-four patients were randomly assigned to the control group, 24 patients received 0.5 μg·kg^− 1^ DEX, and 25 patients received 1.0 μg·kg^− 1^ DEX (Fig. 1). The primary analysis was intention-to-treat (ITT) and involved all patients who were randomly assigned. The loading dose of DEX was not fully administered to one patient because bradycardia occurred in the 1.0 μg·kg^− 1^ DEX group. There were no significant differences among the baseline and preoperative data (Table 1).

**Table 1:** Patient characteristics and preoperative data before receiving drugs in the operating room

	Control group (***n*** = 24)	0.5 μg·kg^**− 1**^ DEX group (***n*** = 24)	1.0 μg·kg^**− 1**^ DEX group(***n*** = 25)	***P***
**Weight (kg)**	62.5 ± 9.0	61.0 ± 9.8	62.2 ± 9.0	0.805
**Height (cm)**	166.9 ± 6.9	166.3 ± 7.4	166.9 ± 6.4	0.932
**Age (yr)**	40.9 ± 7.4	40.0 ± 11.4	42.9 ± 11.8	0.748
**Sex (female)**	13 (54.1%)	12 (50.0%)	12 (48.0%)	0.912
**BIS**	96.7 ± 1.4	96.3 ± 1.6	97.0 ± 1.7	0.095

Data are presented as the means ± SD or numbers (%). Statistical significance was defined as *P* < 0.05

The propofol requirements for LOC decreased significantly in the DEX groups compared with the control group (ANOVA and LSD multiple comparisons, 1.12 ± 0.33 mg·kg^− 1^ for the 0.5 μg·kg^− 1^ DEX group vs. 1.79 ± 0.39 mg·kg^− 1^ for the control group; difference, 0.68 mg·kg^− 1^ [95% CI, 0.49 to 0.87]; *P* = 0.0001) (0.77 ± 0.27 mg·kg^− 1^ for the 1.0 μg·kg^− 1^ DEX group vs. 1.79 ± 0.39 mg·kg^− 1^ for the control group; difference, 1.02 mg·kg^− 1^ [95% CI, 0.84 to 1.21]; *P* = 0.0001), and the propofol requirements for LOC was lower in the 1.0 μg·kg^− 1^ DEX group than that in the 0.5 μg·kg^− 1^ DEX group (ANOVA and LSD multiple comparisons, 0.77 ± 0.27 mg·kg^− 1^ vs. 1.12 ± 0.33 mg·kg^− 1^; difference, 0.34 mg·kg^− 1^ [95% CI, 0.16 to 0.54]; *P* = 0.0001). At the time of LOC, the BIS value was higher in the DEX groups compared with the control group (ANOVA and LSD multiple comparisons, 67.5 ± 3.5 for the DEX 0.5 μg·kg^− 1^ group vs. 60.5 ± 3.8 for the control group; difference, 7.04 [95% CI, 4.85 to 9.23]; *P* = 0.0001) (68.0 ± 4.1 for the 1.0 μg·kg^− 1^ DEX group vs. 60.5 ± 3.8 for the control group; difference, 7.58 [95% CI, 5.41 to 9.75]; *P* = 0.0001). However, there was no difference between the two DEX groups (ANOVA, LSD multiple comparisons, *P* = 0.621). The time to LOC induced by propofol in both the 0.5 μg·kg^− 1^ and 1.0 μg·kg^− 1^ DEX groups was significantly shorter than that in the control group (ANOVA and LSD multiple comparisons, both *P* = 0.0001), and the time to LOC induced by propofol in the 1.0 μg·kg^− 1^ DEX group was significantly shorter than that in the 0.5 μg·kg^− 1^ DEX group (ANOVA and LSD multiple comparisons, *P* = 0.0001) (Tables 2 and 3). No patients in any of the groups developed hypoxia (SpO_2_ < 90%), hypotension or abnormal movements during the study period.

**Table 2:** ANOVA of the main results at LOC induced by propofol

	Control group(***n*** = 24)	0.5 μg·kg^**− 1**^ DEX group (***n*** = 24)	1.0 μg·kg^**− 1**^ DEX group (***n*** = 25)	***P***
**Propofol requirements (mg·kg**^**− 1**^**)**	1.79 ± 0.39	1.12 ± 0.33	0.77 ± 0.27	0.0001
**BIS**	60.5 ± 3.8	67.5 ± 3.5	68.0 ± 4.1	0.0001
**Time to LOC (s)**	309 ± 28	209 ± 53	131 ± 40	0.0001

Data are presented as the means ± SD. Statistical significance was defined as *P* < 0.05

**Table 3:** LSD multiple comparison tests of the main results at LOC induced by propofol

	Propofol requirements (mg·kg^**− 1**^)	BIS values	Time to LOC (s)
	Difference (95% CI) *P*	Difference (95% CI) *P*	Difference (95% CI) *P*
**Control group vs. 0.5 μg· kg**^**− 1**^**DEX group**	0.68 (0.49 to 0.87) 0.0001	−7.04 (− 9.23 to − 4.85) 0.0001	99.91 (76.20 to 123.63) 0.0001
**Control group vs. 1.0 μg·kg**^**− 1**^**DEX group**	1.02 (0.84 to 1.21) 0.0001	−7.58 (−9.75 to −5.41) 0.0001	177.56 (154.08 to 201.03) 0.0001
**0.5 μg·kg**^**− 1**^**DEX group vs. 1.0 μg·kg**^**− 1**^**DEX group**	0.34 (0.16 to 0.54) 0.0001	−0.54 (− 2.71 to1.63) 0.621	77.64 (54.17 to 101.12) 0.0001

Statistical significance was defined as *P* < 0.05

## Discussion

In this study, we administered a loading dose of DEX before the infusion of propofol for anesthesia induction. The propofol requirements for LOC and the BIS value at LOC were measured. Our study showed that DEX facilitated LOC induced by propofol but increased the BIS value at LOC. The propofol requirements for LOC both decreased in the 0.5 μg·kg^− 1^ and 1.0 μg·kg^− 1^ DEX groups when compared with the control group. The result was similar to some early studies in which DEX was considered to decrease the propofol dose during anesthesia [5, 17]. The results indicated that synergistic effect existed between propofol and DEX when they were co-used for sedation. Norepinephrine release in the preoptic area of hypothalamus decreased by DEX makes the disinhibition of the GABAergic and galanergic inhibitory projections to the major arousal nuclei in the midbrain and pons and decreased noradrenergic signaling by DEX acted at the thalamus and cortex both can induce sedation and LOC [18]. Propofol produces sedation by potentiating the activity of GABA_A_ receptors and inhibiting the NMDA-mediated excitatory neurotransmission [19, 20]. Thus, LOC induced by DEX combined with propofol displays a synergistic effect.

In the study, both BIS and clinical evaluation were used to measure the sedative depth. BIS is generally considered a reliable method to detect the level of sedation induced by some hypnotics, especially in propofol anesthesia. However, it had been reported that the BIS values can be influenced by different hypnotic drugs or their combinations [21, 22]. Our results showed the BIS value at LOC was higher when adding DEX to propofol than propofol administration alone. The former study demonstrated that the BIS values was less in DEX sedation than propofol sedation [22]. However, BIS values were higher at LOC when opioid combined with propofol [23]. One possible explanation is that the BIS value was dependent on the dose of propofol, and the BIS value is also larger when small dosage of propofol was administered. Other reasons may include that propofol mainly produces a delta to beta-frequency band in EEG [24], which was quite different from the delta, alpha, range activity induced by DEX [25]. What’s more, the action site of DEX is different from that of propofol [18], which has been considered one factor that influences the BIS value. Furthermore, it should be noted that the BIS value has a time delay between 24 (7) and 122 (23) s [16, 26], which may influence the precision of the measurement of BIS values.

It is quite common that the circulatory and respiratory system were inhibited by propofol infusion. However, there were no episodes of hypotension or hypoxemia in the control group, which might have been due to either the small dosages of propofol than the recommended dosage used in this study or the small number of participants in our study. During the infusion of DEX, one patient exhibited adverse events of severe bradycardia with an HR of 43 beats·min^− 1^. The reason for these events was most likely due to the sympatholytic effect of DEX, a common side effect of α_2_-adrenergic agonists, especially administered with the infusion of loading doses of DEX. However, it could be easily prevented and treated after the administration of receptor-M antagonists. The patient was treated by 0.01 mg·kg^− 1^ atropine, and then the patient’s heart rate rose to over 50 beats·min^− 1^ in a minute. Although the number of participants was small, the sample size was calculated by a statistical tool.

## Conclusions

In conclusion, the pre-administration of 0.5 μg·kg^− 1^ or 1.0 μg·kg^− 1^ DEX could reduce the requirements of propofol for LOC. DEX pre-administration increased the BIS value at LOC induced by propofol.

## Supplementary information

**Additional file 1.** Raw data on oxygen saturation (SpO_2_), mean arterial pressure (MAP) and heart rate (HR).

## Data Availability

The datasets used and/or analyzed during the current study are available from the corresponding author on reasonable request.

## Abbreviations

DEX: Dexmedetomidine
LOC: Loss of consciousness
BIS: Bispectral index
ASA: American society of anesthesiologists
BMI: Body mass index
MAP: Mean arterial pressure
HR: Heart rate
SpO_2_: Peripheral oxygen saturation
ITT: intention-to-treat
